# Role of serotonin on the intestinal mucosal immune response to stress-induced diarrhea in weaning mice

**DOI:** 10.1186/s12876-017-0634-5

**Published:** 2017-06-21

**Authors:** Yulan Dong, Yanan Han, Zixu Wang, Zhuoming Qin, Chenyu Yang, Jing Cao, Yaoxing Chen

**Affiliations:** 1 Laboratory of Veterinary Anatomy, College of Animal Medicine, China Agricultural University, Haidian, Beijing, 100193 People’s Republic of China; 2 Institute of Poultry, Shandong Academy of Agricultural Sciences, Jinan, 250100 China

**Keywords:** Serotonin, Stress diarrhea, Intestinal mucosal immune

## Abstract

**Background:**

During weaning, babies and young animal often experience diarrhea from food intolerance and/or decreasing levels of maternal antibodies, and diarrhea tends to be particularly severe during the early-weaned period, which often exhibits an underdeveloped immune system, a disturbed gut environment and results in nutrient malabsorption and dehydration. It was deduced that neuroendocrine might have close relation with diarrhea, especially 5-HT.

**Methods:**

To explore the role of serotonin (5-HT) in weaning mice subjected to stress-induced diarrhea, 21-day-old weaned mice were divided into the following groups: control group, stress-induced diarrhea group (restrained by binding the hind limbs and intragastric administration of folium sennae with 0.4 g/mL, 15 mL/kg body weight) and para-chlorophenylalanine (PCPA) + stress-induced diarrhea group (30 mg/mL, 300 mg/kg body weight PCPA intraperitoneal injection before stress-induced diarrhea treatment).

**Results:**

Based on results from enzyme-linked immunosorbent assays, histological staining, lymphocyte proliferation assays and flow cytometry analysis, we found that the mice experienced increases in several stress markers, which coincided with severe diarrhea and an increase in 5-HT levels. However, pre-treatment with PCPA resulted in a decrease in the stress indicators and the severity of diarrhea, which correlated with decreased 5-HT levels. Interestingly, stress-induced diarrhea caused changes in various aspects of the immune system, including the amount of intraepithelium lymphocytes, CD4^+^/CD8^+^ T lymphocyte populations, B and T lymphocyte proliferation, and the secretion of sIgA and cytokines in the small intestine and ileum. However, these immune system changes could be reversed upon treatment with PCPA.

**Conclusions:**

We observed a distinct correlation between 5-HT levels and the occurrence of stress-induced diarrhea in weaning mice, which may result in the deregulation of the mucosal immune system.

## Background

The World Health Organization (WHO) reported that diarrhea was the second leading cause of death in children under 5 years of age in 2013. Moreover, the WHO estimates that approximately 1.7 billion cases of diarrhea occur every year worldwide [1]. During weaning, babies often experience diarrhea from food intolerance and/or decreasing levels of maternal antibodies [2]. Diarrhea is the manifestation of a disturbed gut environment and results in nutrient malabsorption and dehydration [3]. Weaning is one of the most critical periods during which the infant is exposed to several stressors, including nutritional, social and environmental changes [4–6]. Not only infants, but also piglets are sensitive to weaning which often exhibits an underdeveloped immune system, digestive disorders and post-weaning diarrhea [7]. So, piglet is a perfect model of weaning diarrhea. Weaning piglet from the sow contributes to intestinal and immune system dysfunction and results in reduced pig health, growth, and food intake, particularly during the first week after weaning [4].

The mechanism of stress is complex and typically triggers two endocrine systems: (1) the hypothalamic-pituitary-adrenal (HPA) axis resulting in glucocorticoid production and (2) the sympathetic nervous system resulting in catecholamine release (epinephrine and norepinephrine). The HPA axis and serotonin (which is also known as 5-hydroxytryptamine (5-HT)) are closely cross-regulated in mammals under normal physiological conditions [8]. In the gastrointestinal tract, which is the largest endocrine organ, 5-HT plays a major role as a neurotransmitter molecule. Ninety-five percent of the 5-HT in the body is synthesized, stored, and released by enterochromaffin (EC) cells, which are a subset of enteroendocrine cells that reside within the intestinal mucosa [9–11]. EC cells constitute part of the epithelial lining of the gut and are found near enteric neurons in the submucosal and myenteric plexuses. However, the release of 5-HT from the EC cells is not always beneficial. Exogenously administered 5-HT increases fecal pellet output in rats and causes diarrhea in mice [12–14]. Therefore, 5-HT has been proposed to mediate bowel dysfunction caused by stress. 5-HT mediates GI peristalsis, secretion, vasodilation and perception of pain or nausea by activating a diverse family of 5-HT receptors on the intrinsic and extrinsic afferent nerve fibers located within the lamina propria. Porcine GI mucosa predominantly expresses the 5-HT4 receptor splice variant, suggesting that this receptor plays a large role in 5-HT-mediated mucosal effects [15]. The intestinal mucosal immune barrier provides an important defensive barrier against intestinal diarrhea. Recent studies have revealed that the synthesis and transport of 5-HT in immune cells are much more diverse than previously realized [16]. Many 5-HT immunoreactive cells are in contact with or are in very close proximity to CD3+ and CD20+ lymphocytes within the epithelium of the intestines of rhesus monkeys (*Macaca mulatta*), which further supports a possible role for 5-HT in the gut mucosal immune response [17]. Furthermore, antagonism of the 5-HT1B receptor inhibits the proliferation of both human and murine primary helper T cells as well as the proliferation of human helper T cell lines [18].

A recent review by Gershon noted that enteric 5-HT is a polyfunctional signaling molecule that acts as a neurotransmitter paracrine factor, an endocrine hormone and a growth factor in developing and mature animals [19]. Nonetheless, despite abundant evidence on the functions of 5-HT, the effect of 5-HT on the development of diarrhea remains unclear. To explore the mechanisms associated with 5-HT on weaning stress-induced diarrhea disease, 21-day-old weaned mice were treated with para-chlorophenylalanine (PCPA), which inhibits the activity of tryptophan hydroxylase, a rate-limiting component of 5-HT synthesis, resulting in reduced 5-HT synthesis. Additionally, we examined various mucosal immunity parameters in these mice.

## Methods

### Animal treatment

A total of 204 male CD1 mice (weaned at 21 d; Vital River Laboratory Animal Technology Co. Ltd., Beijing, China) were housed under standard conditions (temperature of 21 ± 1 °C, relative humidity of 50 ± 10%) with a regular 12 h light: 12 h dark cycle. The mice were separated into 3 groups (*n* = 68). The first group of mice was subjected to stress-induced diarrhea by treating the mice with folium sennae (0.4 g/mL, 15 mL/kg body weight) via intragastric administration 4 h after intraperitoneal injection of sterile saline. The second group was the PCPA + stress-induced diarrhea group, which was treated by intraperitoneal injection of sterile saline containing PCPA (30 mg/mL, 300 mg/kg body weight) followed by the intragastric administration of folium sennae (0.4 g/mL, 15 mL/kg body weight). After the mice were treated, both groups were restrained by binding the hind limbs, leaving the fore limbs unbound, for 1 h. The third group was the control, which was treated with sterile saline for a period of 5 d. Blood glucose levels (mmol/L) were measured by bleeding the tail vein to glucometer (GC14906201, ROCHE), then they were sacrificed by cervical dislocation after the mice were anesthetized using 2% pentobarbital (4 mL/kg). All animal procedures were approved by the China Agricultural University Institutional Animal Care and Use Committee (XK20110915).

### Plasma and tissue preparation

Plasma samples were collected for the detection of corticosterone (CORT) and 5-HT. The intestinal segments were fixed in 4% paraformaldehyde and then embedded in paraffin for histological analysis. Portions of the intestinal segments were rapidly homogenized, and clarified lysates were obtained by centrifugation (1000 x g for 20 min). The tissue extracts were stored at −80 °C for cytokine and 5-HT analysis. Some intestinal segments were designated for the evaluation of lymphocyte proliferation ability as well as flow cytometry analysis of the lymphocyte isoforms.

### Enzyme-linked immunosorbent assay

The protein concentrations of the tissue lysates were determined using a bicinchoninic acid (BCA) (mg/mL) prior to enzyme-linked immunosorbent assay (ELISA) analysis. The CORT concentration in the plasma was measured using a competitive ELISA (CEA540Ge, Uscn Life Science Inc., Wuhan, China). All of the tests were performed according to the manufacturers’ instructions. The range of detection for this assay ranged from 6.17 to 500 ng/mL, and the final concentration is presented in μg/g protein. The sIgA and 5-HT levels in the plasma and intestinal tissue were measured using a biotin-labeling double-antibody sandwich enzyme-linked immunosorbent assay (DZE21316, LBTR-EL-1553, Beijing Limbo Terry Technology Development co., Ltd., Beijing, China). The range of 5-HT detection for this ELISA assay ranged from 10 to 3000 ng/L, and the final concentrations are presented in ng/g protein. TNF-α (EK0527), IL-2 (EK0398), IL-10 (EK0417), and IL-4 (EK0405) levels were evaluated using a biotin-labeling double-antibody sandwich enzyme-linked immunosorbent assay (Wuhan Boster Biological Engineering Co., Ltd., Wuhan, China), and the range of cytokine detection for the assay ranged from 15.6 to 1000 pg/mL. The concentrations of the cytokines are presented in pg/mg protein.

### Histological staining of intraepithelium lymphocytes in the intestines

Tissue sections (5 μm) were mounted onto gelatinized glass slides and then deparaffinized and rehydrated. Tissue sections were stained with hematoxylin and eosin for the intraepithelium lymphocyte **(**IEL) test. Five tissue sections were evaluated for each mouse. IEL cells were counted by microscopy per 100 epithelial cells.

### Lymphocyte isolation

Mice were sacrificed, the intestine or spleen, peyer’s patch were rapidly removed, sliced into small pieces and incubated with 0.1% collagenase at 37 °C for 60 min. The digestive tissues were passed through a nylon tissue sieve (200 nylon mesh per 2.5 cm) and washed twice with RPMI-1640 medium (Gibco-BRL, Grand Island, NY, USA). After the centrifugation, the precipitate was suspended in 3 mL RPMI 1640 complete medium and combined with 3 mL lymphocyte separation medium (LTS1077, Tianjin Haoyang Biologicals Technology Co., Ltd., Tianjin, China) for centrifugation (800×g, 10 min). The isolated lymphocytes viability (>98%) was detected by 0.5% trypan blue dye, and the cell suspensions were cultured in 1 mL RPMI-1640 at a density of 1X10^6^ cells/mL.

### Lymphocyte proliferation ability assay

Lymphocyte suspensions (200 μL) were seeded into a 96-well flat bottom plate (Costar 3599, Corning Inc., NY, USA) and cultured in the presence of 15 μg/mL LPS (L2630, Sigma) or ConA (C2272, Sigma). After incubating the lymphocytes at 37 °C in a humidified 5% CO_2_ incubator for 68 h, the proliferation index was determined using 5 mg/mL methyl thiazolyl tetrazolium (MTT, Sigma). Control cells were incubated with RPMI-1640 alone. Plates were incubated for 4 h after the addition of 10 μL MTT per well. Then, 90 μL of 10 mg/mL sodium dodecyl sulfate (SDS, Sigma) solution was added to each well. Finally, the microplate was read on a BioRad microplate reader (Model 550) at a wavelength of 570 nm after being incubated for 4 h. The proliferation of lymphocytes was expressed as the stimulation index (SI) according to the following equation: Stimulation index (SI) = OD570 (stimulated cells)/OD570 (unstimulated cells).

### Flow cytometry analysis

Flow cytometry was used to determine the phenotype of the lymphocytes. Isolated lymphocytes were stained for 30 min in the dark at room temperature with 1 μL of monoclonal antibodies specific for T cell surface markers. These antibodies included APC-conjugated anti-CD3 (553,066, BD Biosciences, San Diego, USA), FITC-conjugated anti-CD4 (553,046, BD Biosciences, San Diego, USA) and PE-conjugated anti-CD8 (553,032, BD Biosciences, San Diego, USA). Then, the lymphocytes were washed with 0.01 M PBS (0.2 mL) and centrifuged at 500×g for 5 min; this step was repeated for a total of three washes. After the final wash, the cells were resuspended in 0.2 mL of PBS and subjected to flow cytometry analysis. The lymphocytes were analyzed on a FACSCalibur flow cytometer (BD Biosciences) using the CellQuest program (BD Biosciences).

### Statistical analysis

Data were expressed as the means ± SD. Data were analyzed using SPSS version 11.5 software. One-way ANOVA was used for statistical analyses. Differences corresponding to *P* < 0.01 were considered very significant, and differences corresponding to *P* < 0.05 were considered significant. P ≈ 0.000 was represented as *P* < 0.001.

### Ethical approval and consent

All animals received humane care and all animal procedures in this study were performed in accordance with the Guidelines for Animal Experimentation of China Agriculture University.

## Results

### Severity of diarrhea

**Fig. 1:**
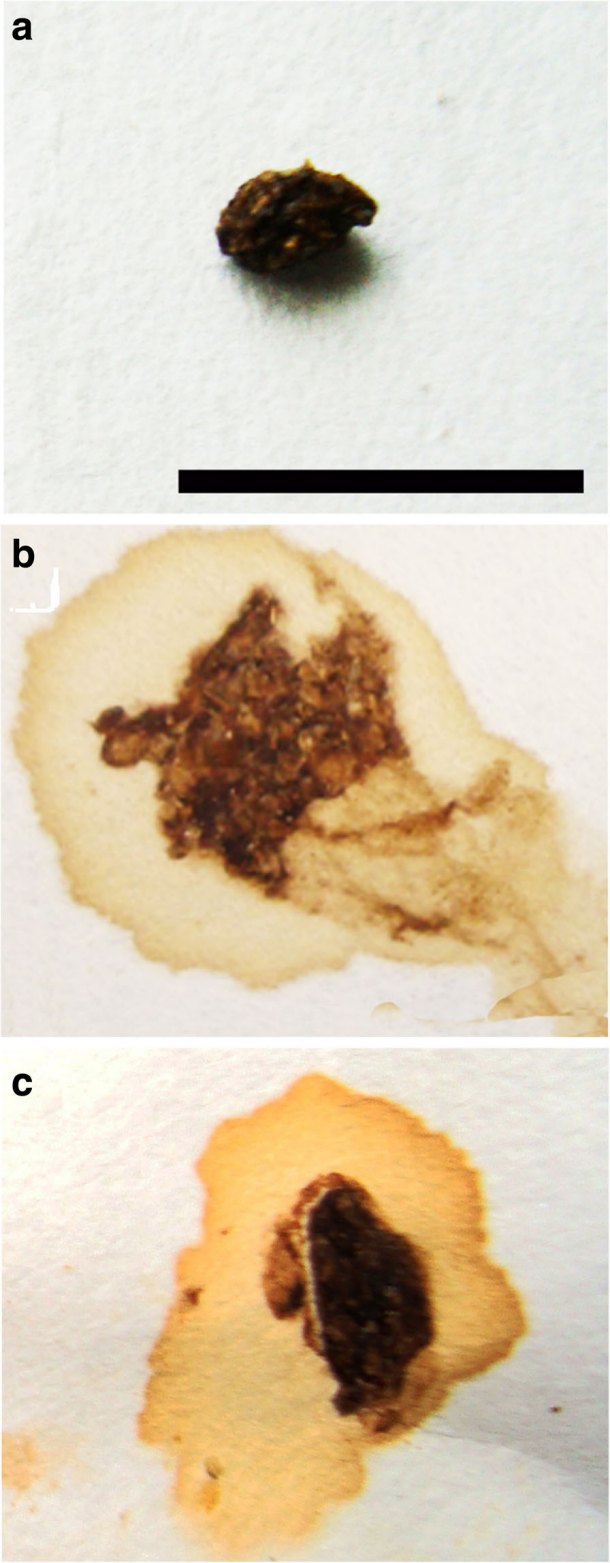
The stools from mice in the different treatment groups. **a** Control group; **b**: Stress-induced diarrhea group; and **c**: PCPA + Stress-induced diarrhea group. The stool of control mice showed dry and normal granulous texture **a**. Mice experienced severe diarrhea after being stressed physically and with folium sennae treatment **b**, but the signs of diarrhea improved in mice pre-treated with PCPA **c**. Scale bar = 1 cm in **a**, **b** and **c**

The stool in the control group presented with a normal granulous appearance. However, the stress-induced diarrhea group, which was treated with physical stress and folium sennae, developed serious diarrhea with soil in the anus. In the mice that were pre-treated with PCPA, slight diarrhea was observed (Fig. 1).

### The measurement of stress activity

Blood glucose and CORT levels are used as markers to indicate the stress level in the body (Table 1). The blood glucose (*P* = 0.002) and CORT (*P* = 0.015) levels in the group of mice subjected to stress-induced diarrhea increased significantly when compared with the control mice. However, the levels of blood glucose and CORT in the mice pre-treated with PCPA and then subjected to stress-induced diarrhea showed slightly decreased levels of blood glucose and CORT when compared with the untreated stress-induced diarrhea group. Therefore, stress-induced diarrhea coincided with increased indicators of stress activity in the mice, which could be partially reversed upon pre-treatment with PCPA.

**Table 1:** Changes in the blood glucose, cortisol and 5-HT levels of the mice in the different groups (mean ± SD)

Index	Tissue	Control	Stress diarrhea	PCPA + Stress diarrhea
Blood glucose (mmol/L)	Plasma	9.61 ± 0.34^a^	10.74 ± 0.65^b^	10.08 ± 0.51^ab^
Cortisol (μg/g.prot)	Plasma	1.91 ± 0.42^a^	2.80 ± 0.16^b^	2.55 ± 0.13^b^
5-HT (ng/g.prot)	Plasma	4.48 ± 0.34^b^	7.28 ± 0.92 ^c^	3.52 ± 0.29^a^
	Duodenum	21.93 ± 1.74^a^	29.25 ± 1.55^b^	23.43 ± 1.79^a^
	Jejunum	48.07 ± 4.41^a^	73.47 ± 6.46^c^	57.05 ± 7.07^b^
	Ileum	50.35 ± 7.17^ab^	78.40 ± 8.22^c^	54.56 ± 3.83^b^
	Colon	74.25 ± 7.52^b^	94.75 ± 16.08^c^	49.11 ± 4.79^a^

Note: Different letters in the same line indicate significant differences between the treatments (*P* < 0.05); the same letter in the same line of data means that there was no significant difference between the groups (*P* > 0.05)

### Changes of 5-HT concentration associated with diarrhea and stress activity

To explore the relationship between diarrhea and 5-HT levels and to determine the effects of PCPA on 5-HT secretion, 5-HT levels in the different groups were determined (Table 1). 5-HT levels in the plasma and the intestinal tissue of the stress-induced diarrhea group increased relative to the control group (*P* < 0.001). Moreover, PCPA significantly reversed the increase in 5-HT levels in mice subjected to stress-induced diarrhea (*P* < 0.001).

### The number of IELs in intestine

**Fig. 2:**
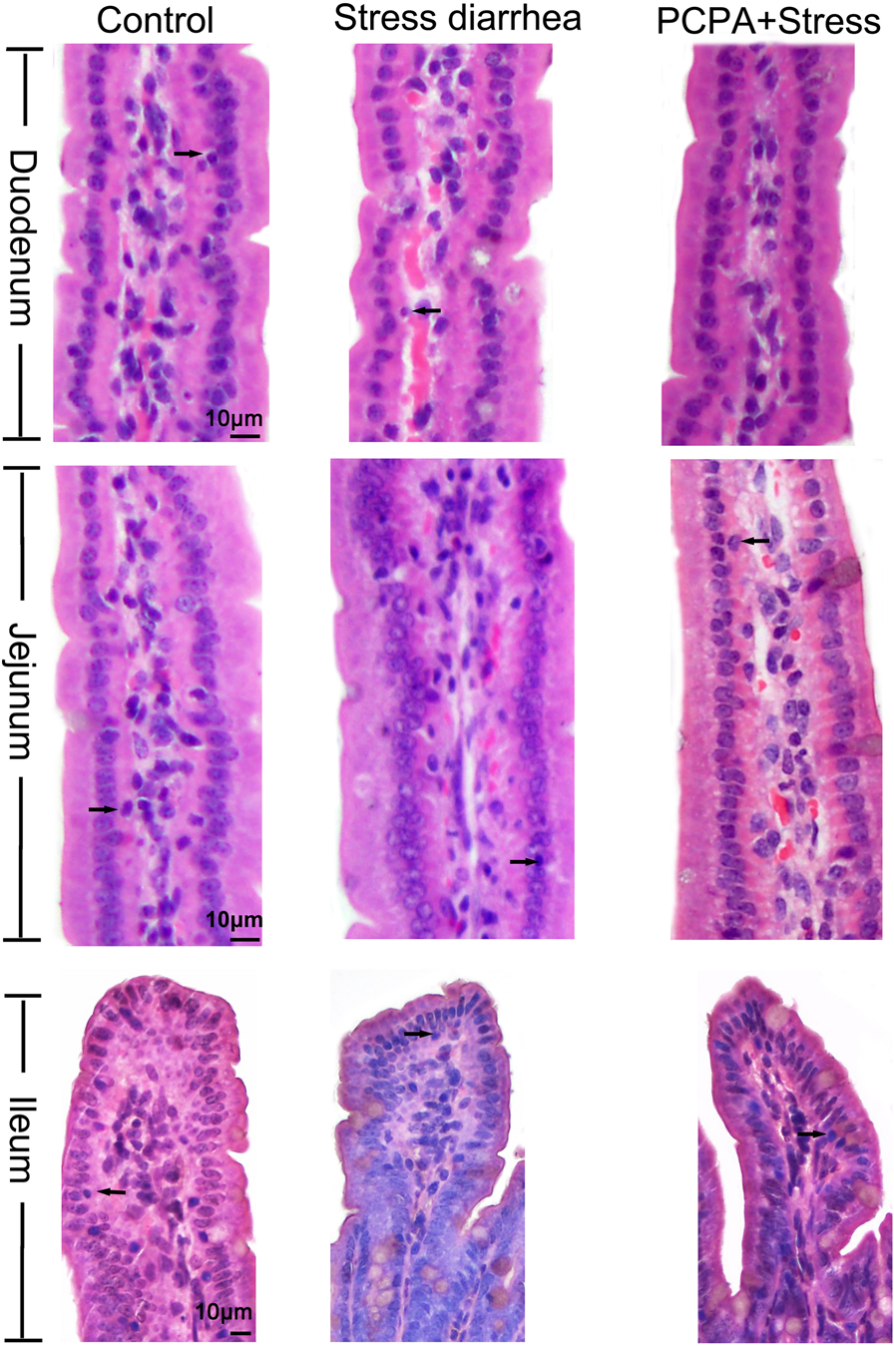
The morphology of IELs in the small intestine of mice (H&E staining). The arrow shows an IEL, which is small, and contains a large and round nucleus. The depth of the stain displays scattered distribution of IELs among the intestinal villus columnar epithelium, and most of the IELs are situated around the epithelial basement membrane; few IELs can be observed near the free surface epithelial cells. The scale bar represents 10 μm in the duodenum and jejunum, and the scale bar represents 10 μm in the ileum

**Fig. 3:**
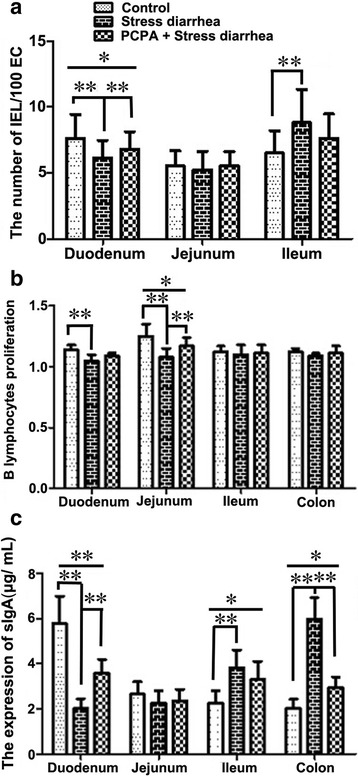
The changes in the levels of IELs and B lymphocytes in mice. **a**. The changes in the intraepithelium lymphocyte (IEL) amount per 100 epithelial cells of the small intestine in the different treatment groups are depicted. Stress-induced diarrhea resulted in reduced amounts of IELs in the duodenum and jejunum but an increased amount of IELs in the ileum. PCPA pre-treatment reversed the effect of stress-induced diarrhea on the IEL populations. **b**. The changes in the B lymphocyte proliferation in the intestines of the different treatment groups are depicted. The changes in the B lymphocyte population in the duodenum and jejunum displayed a similar trend to the IEL populations in the duodenum; however, no differences were observed in the B lymphocyte population in the ileum and colon. **c**. The sIgA levels represent the B lymphocyte immune activity. *p < 0.05, **p < 0.01

IELs appeared small, whereas the nuclei appeared large and round. The results of the staining showed a scattered distribution of IELs among the intestinal villus columnar epithelial cells, most of which were situated around the epithelial basement membrane. A few IELs were observed near the surface epithelial cells (Fig. 2). Data analysis showed that stress-induced diarrhea resulted in reduced amounts of IELs in the duodenum (*P* < 0.001) and jejunum (*P* = 0.223) but an increased amount of IELs in the ileum (*P* < 0.001). PCPA pre-treatment reversed the effect of stress-induced diarrhea on the IEL populations (Fig. 3a).

### The detection of B lymphocyte proliferation and sIgA levels in intestine

Our laboratory previously found that a concentration of 15 μg/mL LPS could effectively stimulate B lymphocyte proliferation and transformation. When compared with the control group, the stress-induced diarrhea group showed a decreased B lymphocyte proliferation index in the duodenum (*P* = 0.009) and jejunum (*P* < 0.001). PCPA pre-treatment of the mice subjected to stress-induced diarrhea resulted in an increase in the B lymphocyte proliferation index in the duodenum (*P* = 0.059) and jejunum (*P* = 0.007). However, the proliferation index in the jejunum of the PCPA-pre-treated mice remained lower than the control group (*P* = 0.016). We observed no significant differences in the B lymphocyte proliferation index in the ileum and colon among the three groups (Fig. 3b).

The concentrations of sIgA in the duodenum and the colon were increased in the stress-induced diarrhea group relative to the control mice (Fig. 3c). When compared with the control group, the stress-induced diarrhea group showed significantly reduced sIgA levels in the duodenum (*P* < 0.001) but significantly increased sIgA levels (*P* < 0.001) in both the ileum and the colon. The PCPA + stress-induced diarrhea group showed a significant increase in the concentration of sIgA in the duodenum (*P* < 0.001) relative to the stress-induced diarrhea group, but these levels remained significantly lower (*P* < 0.001) than those of the control group. The concentrations of sIgA in the ileum (*P* = 0.183) and colon (*P* < 0.001) of the PCPA + stress-induced diarrhea group was reduced relative to the stress-induced diarrhea group, but these levels were still significantly higher than those of the control group (*P* < 0.05). Therefore, stress-induced diarrhea affected the secretion of sIgA in the intestines, and PCPA could partially reverse the altered secretion profile.

### The influence of 5-HT on T lymphocyte proliferation and CD4^+^/CD8^+^ T lymphocytes

**Fig. 4:**
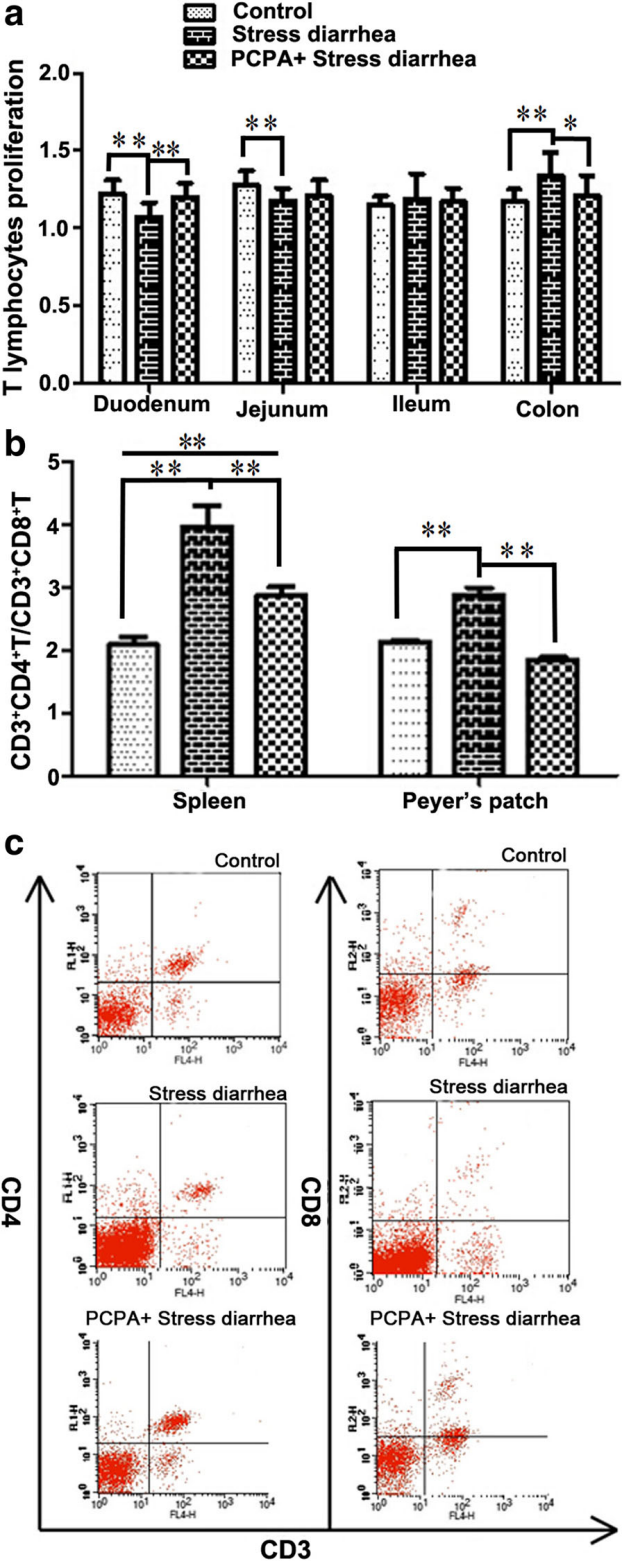
The changes in T lymphocyte proliferation and subsets number in mice. **a** The changes in the T lymphocyte proliferation in the intestines for the different treatment groups are depicted. In the duodenum and colon of the mice subjected to stress-induced diarrhea showed increased T lymphocyte populations when compared with the control group; however, the increase in T lymphocytes was reversed upon pretreatment with PCPA. **b** The changes in the CD3^+^CD4^+^/CD3^+^CD8^+^ T lymphocyte populations in the spleen and Peyer’s patch for the different treatment groups. **c** Flow cytometry was performed to identify the CD3^+^CD4^+^ and CD3^+^CD8^+^ T lymphocytes in the spleens of the different groups. *p < 0.05, **p < 0.01

Our laboratory had found previously that 10 μg/mL ConA could effectively stimulate T lymphocyte proliferation and transformation. Compared with the control group, in the stress-induced diarrhea group, the T lymphocyte proliferation index in the duodenum (*P* < 0.001) and jejunum (*P* = 0.006) decreased significantly but increased significantly in the colon (*P* = 0.009). The T lymphocyte proliferation index of the PCPA + stress-induced diarrhea group increased in the duodenum (*P* < 0.001) relative to the stress-induced diarrhea group but did not reach the levels of the control mice (Fig. 4a).

The number of T lymphocytes was measured by flow cytometry to explore the relationship between stress-induced diarrhea, 5-HT and T lymphocyte immunity. The ratio of CD3^+^ CD4^+^ T cells and CD3^+^ CD8^+^ T cells of splenic and PP node origin were shown (Fig. 4b). The ratio of CD3^+^ CD4^+^ T cells/CD3^+^ CD8^+^ T cells in the spleen (*P* < 0.001) and PP (*P* < 0.001) of the stress-induced diarrhea group were higher than the control group. However, it was reduced in the spleen (*P* < 0.001) and PP (*P* < 0.001) of the PCPA + stress-induced diarrhea group relative to the stress-induced diarrhea group. We observed no difference in the CD3^+^ CD8^+^ T cell counts among the three groups, suggesting that CD3^+^ CD4^+^ T cells but not CD3^+^ CD8^+^ T cells were affected by stress-induced diarrhea and PCPA treatment. Flow cytometry was performed to identify the CD3^+^CD4^+^ and CD3^+^CD8^+^ T lymphocytes in the spleens of the different groups (Fig. 4c).

### The influence of 5-HT on cytokines concentration in intestine tissue homogenate

**Fig. 5:**
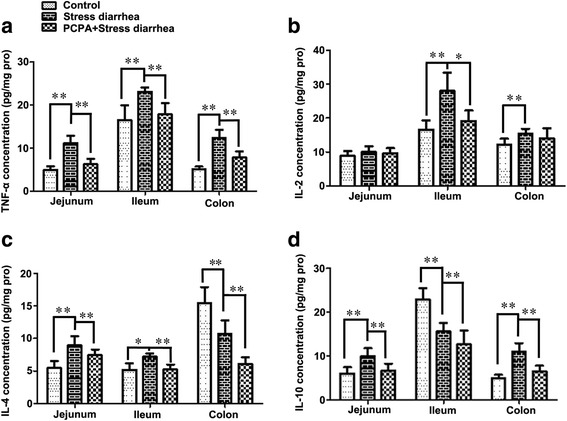
The changes in the cytokines in the intestines of mice. Cytokine levels were determined using ELISA. TNF-α (**a**) and IL-2 (**b**) levels increased in the the jejunum and the colons of mice subjected to stress-induced diarrhea when compared with the control group. However, IL-4 levels (**c**) in the colon and IL-10 levels (**d**) in the ileum decreased sharply due to stress-induced diarrhea. Pretreatment with PCPA caused a decrease in the levels of the four cytokines when compared with the stress-induced diarrhea group. *p < 0.05, **p < 0.01

The concentrations of TH1 and TH2 cytokines in the jejunum of the stress-induced diarrhea group were increased relative to the control group (Fig. 5). However, the concentration of cytokines decreased to normal levels in the PCPA + stress-induced diarrhea group. Relative to the untreated stress-induced diarrhea group, TH1 cytokines (TNF-α, *P* < 0.001; IL-2, *P* = 0.833) were reduced in the PCPA + stress-induced diarrhea group to a level similar to that of the control mice. Similarly, the numbers of TH2 type cytokines (IL-4, IL-10, *P* < 0.001) of the PCPA + stress-induced diarrhea mice were reduced when compared with the untreated group. The trends in cytokine levels in the ileum were similar to the trends observed in the jejunum in the untreated and PCPA-treated stress-induced diarrhea groups except for IL-10. In the colon, the TNF-α, IL-2 and IL–10 levels in the stress-induced diarrhea group were also increased, but IL–4 levels were reduced when compared with the control group. Furthermore, PCPA treatment reduced TH1 and TH2 cytokine secretion associated with stress-induced diarrhea.

## Discussion

Weaning is one of the most stressful periods for animals. As we know that weaning caused animals to a number of stressors, including a different food sources, a different physical environment (room, building, farm, water supply, etc.), increased exposure to pathogens and environmental antigens, all of these can trigger intestinal, immunological, and behavioral changes in the animal. Many “stress hormones”, such as thyroid hormones, thyroxine and triiodothyronine, and cortisol, are involved in the development of stress-related conditions, which suggests that the activation of the adrenergic axis is also involved in stress-related conditions [20]. In our study, the blood glucose and CORT levels of the mice subjected to stress-induced diarrhea increased significantly when compared with the control mice, which suggests a close relationship between stress and diarrhea. The increase of serum CRF and cortisol levels in weaned pigs indicated that weaning induces the activation of stress pathways, which can result in intestinal dysfunction [21].

However, the levels of blood glucose and CORT in mice treated with PCPA and subjected to stress-induced diarrhea decreased slightly compared with untreated mice subjected to stress-induced diarrhea. Numerous stressors can increase 5-HT synthesis/turnover [22, 23]. For example, DSS-colitis increases 5-HT levels by increasing the amount of EC cells and/or increasing the content of 5-HT in the EC cells [24]. Severe diarrhea was accompanied by an increase in 5-HT levels in our study. Moreover, treatment with the tryptophan hydroxylase inhibitor PCPA resulted in a reduction in 5-HT levels and a decrease in the severity of diarrhea. Thus, we observed a direct correlation between 5-HT levels and diarrhea, which is consistent with previous reports suggesting that ramosetron, a 5-HT3 receptor antagonist, can reduce stress-induced diarrhea in rats [25], and improve stool consistency in male patients with IBS-D (irritable bowel syndrome - diarrhea) [26]. However, a decrease in the amount of ileal serotonin cells is associated with visceral hypersensitivity associated with all IBS subtypes [27]. In summary, the role of 5-HT in the context of diarrhea is complex and dubious.

5-HT is one of the most extensively studied neurotransmitters of the central nervous system and has been shown to affect platelets, lymphocytes, monocytes, macrophages, mast cells, pulmonary neuroendocrine cells, enterochromaffin cells of the gut, and other cell types. Mössner and Lesch (1998) reviewed the effects of 5-HT and suggested that 5-HT may mediate the interaction between the nervous and immune systems through four different pathways in addition to the central role of 5-HT in the induction of the emetic reflex [28]. The mammalian intestinal tract is the largest immune organ in the body and is composed of several different types of cells [29]. 5-HT serves as a link between the gut and immune regulation [30, 31]. This suggests that 5-HT may be involved in the nervous system-mediated regulation of the digestion-immune system interface.

In this study, we counted the number of IELs per 100 epithelial cells in the small intestine. When compared with the control group, the number of IELs in the duodenum was decreased but the number of IELs in the ileum was increased of the stress-induced diarrhea group. In one report, patients with IBS-D displayed significantly higher IELs in colon and rectum than the healthy controls [32]. Therefore, diarrhea might induce more strong immunity activity after ileum. In our study, changes in the IEL population of the stress-induced diarrhea groups were reversed upon treatment with PCPA. The B lymphocyte proliferation index and the concentration of sIgA in the duodenum and jejunum showed trends similar to those of the IELs. However, these trends differed in the ileum and colon. The B lymphocyte proliferation index did not change, but the concentration of sIgA was higer in the ileum and colon; these changes were reversed upon pre-treatment with PCPA. In addition, serotonin treatment alone had no effect on splenic cell proliferation but increased mitogen-stimulated B cell proliferation in a dose- and time-dependent manner [33]. But the number of IgA+ cells and the level of sIgA in the intestinal fluid are lower in mice subjected to LPS-induced diarrhea than in the control group [34]. The T lymphocyte proliferation index showed a similar trend with respect to the concentration of sIgA in our study. Inhibition of 5-HT synthesis by pre-treatment with PCPA impairs T-cell activation and proliferation [35]. However, the ratio of CD3^+^ CD4^+^ T cells/CD3^+^ CD8^+^ T cells in the spleen and PP was higher in mice subjected to stress-induced diarrhea when compared with the control group, and PCPA could repair it to the control group level. 5-HT has also been reported to modulate T-cell activation and differentiation [16, 17, 35–37].

These results suggest that stress-induced diarrhea decreases the mucosal immunity in the duodenum and jejunum without affecting the ileum and colon. Furthermore, these results suggest that PCPA pre-treatment ameliorates these pathological signs.

In this study, the TNF-α and IL-2 cytokine levels from the jejunum to the colon were increased in the stress-induced diarrhea group relative to the control group. Increased concentrations of IL-2 and IL-2R in patients relative to control patients have been reported in active IBD lesions [38, 39]. IL-4 and IL-10 are pleiotropic anti-inflammatory cytokines that mainly function by suppressing the pro-inflammatory milieu [40]. The IL-4 and IL–10 concentrations decreased in the colon and ileum in the stress-induced diarrhea group but increased in the jejunum of these mice. These results suggest that stress-induced diarrhea caused most of the inflammation associated with the small intestine and the colon. 5-HT levels also increased in the stress-induced diarrhea group, which correlated with cytokine levels. A previous paper reported that the components of the stress system, including norepinephrine (NE) and glucocorticoids, appear to mediate a Th2 (IL-4 and IL-10) shift, whereas serotonin (5-HT) and melatonin might mediate a Th1 (TNF-α and IL −2) shift [41].

When the stress-induced diarrhea group was pretreated with PCPA, the concentration of pro-inflammatory cytokines (TNF-α and IL-2) returned to normal levels, but the anti-inflammatory cytokines (IL-4 and IL-10) also decreased. These results suggest that low 5-HT levels due to PCPA treatment could inhibit the secretion of all types of cytokines. It was similar to that 5-HT (100 nM-10 μM) significantly increased IL-6 expression of MLO-Y4 cells [42]. However, 5-HT (10^−11^ − 10^−9^ M) stimulated IL-10 and inhibited IL-12 and TNF release of human alveolar macrophages [43]. In brief, a normal level of 5-HT appears to balance the levels of cytokines thus contributing to a healthy microenvironment within the gut.

## Conclusions

Our results suggest a close relationship between 5-HT levels and diarrhea. Diarrhea was accompanied by an increase in 5-HT levels, moreover, a decrease in 5-HT levels was observed upon pre-treatment with PCPA, which correlated with reduced diarrhea severity. The regulatory mechanisms of 5-HT on the development of diarrhea could disturb the balance of the immune system in the gut, as changes in the levels of IELs, CD4^+^/CD8^+^ T lymphocytes, B and T lymphocyte proliferation, secretion of sIgA and cytokines were observed in the intestines of mice subjected to stress-induced diarrhea.

## Abbreviations

5-HT: 5-hydroxytryptamine (serotonin)
ConA: Concanavalin A
CORT: Corticosterone
DSS: Dextran sulfate sodium
EC: Enterochromaffin
ELISA: Enzyme-linked immunosorbent assay
IBS-D: irritable bowel syndrome – diarrhea
IEL: Intraepithelium lymphocyte
IL-10: Interleukin-10
IL-2: Interleukin-2
IL-4: Interleukin-4
LPS: Lipopolysaccharide
MTT: Methyl thiazolyl tetrazolium
NE: Norepinephrine
PCPA: Para-chlorophenylalanine
SDS: Sodium dodecyl sulfate
SI: Stimulation index
TNF-α: Tumornecrosisfactor-α
WHO: World Health Organization
